# Primary osteosarcoma of the urinary bladder following cyclophosphamide therapy for systemic lupus erythematosus: a case report

**DOI:** 10.1186/1752-1947-3-39

**Published:** 2009-01-29

**Authors:** Dilek Ertoy Baydar, Cigdem Himmetoglu, Sertac Yazici, Halil Kiziloz, Haluk Ozen

**Affiliations:** 1 Department of Pathology, Hacettepe University Hospital, Ankara, Turkey; 2 Department of Urology, Hacettepe University Hospital, Ankara, 06100, Turkey

## Abstract

**Introduction:**

The association of systemic lupus erythematosus with malignancies is an uncommon occurrence. We present the case of an osteosarcoma of the urinary bladder developing in a patient with a prolonged history of active systemic lupus erythematosus. This is a previously unreported association. Primary osteosarcoma is an extremely rare disease in the urinary bladder.

**Case presentation:**

A 24-year-old Caucasian woman with a 13-year history of systemic lupus erythematosus, who had been treated with high dose immunosuppressive agents, presented with pain and hematuria. A deeply invasive high-grade tumor was detected in the urinary bladder and the patient underwent radical surgery. A diagnosis of osteosarcoma was made based on the characteristic histology.

**Conclusion:**

Predisposing factors for primary sarcomas in the urinary bladder are mostly unknown; however, in our case, long-term administration of immunosuppressive agents, as well as long standing systemic lupus erythematosus, may both be of significance.

## Introduction

In this report, we present the case of a 24-year-old woman with a primary osteosarcoma of the urinary bladder. Malignant mesenchymal tumors comprise less than 0.04% of urinary bladder malignancies [1]. The most frequent histology is rhabdomyosarcoma in children and leiomyosarcoma in the older age group. In the English language medical literature, only 30 cases of primary osteosarcoma of the urinary bladder have been reported to date. Our case, being the 31st, is unique in respect to the patient's age and the history of systemic lupus erythematosus (SLE), which appears as a possible predisposing factor. The patient had been treated with immunosuppressive medications including cyclophosphamide for active SLE for many years. Neoplastic transformations in SLE are accepted as occurring more frequently than in the general population [2];most tumors are lymphomas with sarcomas being exceptional and, to our knowledge, no previous cases of osteosarcoma, in any location in the body, accompanying SLE, have been reported.

## Case presentation

We first saw our patient in 1995 when she was an 11-year-old girl and she presented with fever, fatigue, loss of appetite, malar rash and swelling in the small joints of her hands. Laboratory investigations at that time revealed elevated antinuclear antibody titer (1/1000) and anti-dsDNA levels (124 IU/mL), anemia, decreased C3 (14.3) and C4 (8.1) and SLE was diagnosed. She was treated with azathioprine 100 mg for 3 months, hydroxychloroquine 400 mg for 9 months and prednisolone 10–32 mg daily. Her disease was in remission for 2 years. In December 1997, cyclophosphamide (50–150 mg/day) was added and hydroxychloroquine was restarted because of flare up of the SLE activity with direct Coombs positive hemolytic anemia and elevated sedimentation rate. Thereafter, there was no complete remission and she was continuously on immunosuppressive medications, with the dose regulations depending on her white blood cell count, and she also needed pulse methylprednisolone administration once a month. She also had frequent urinary tract infections with Gram negative bacteria which were treated with several antibiotics.

**Figure 1:**
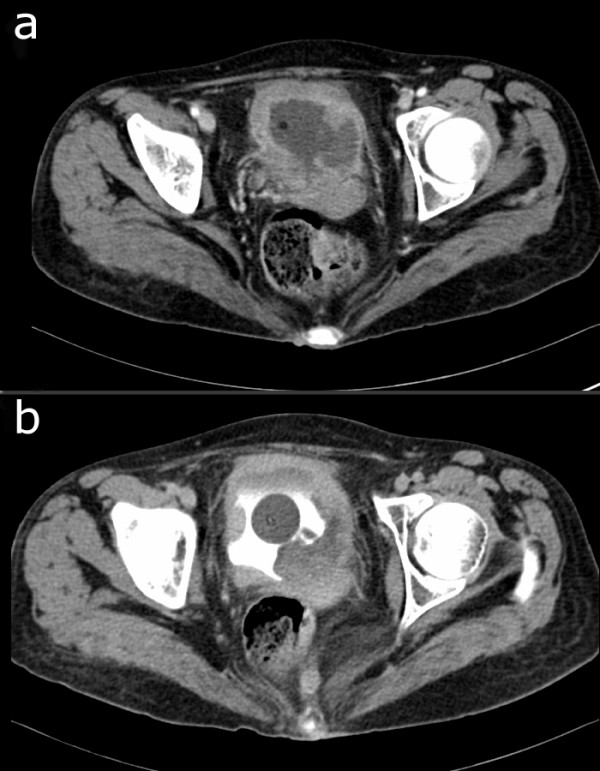
**Computed tomography**. Unenhanced (a) and enhanced (b) CT of pelvis showing diffuse extensive thickening of bladder wall.

In November 2007, she presented again complaining of flank pain and bloody urine. Ultrasonogram revealed left-sided hydronephrosis and dilatation of the left ureter and a nephrostomy catheter was placed in her left renal pelvis. Cystoscopic examination was performed and a polypoid lesion in the bladder obstructing the left ureteral orifice was observed. Computerized tomography (CT) of the pelvis indicated diffuse thickening of the bladder wall throughout the organ and obscured fat planes between the bladder and uterus, arousing suspicion of perivesical tissue invasion by a malignancy (Figures 1a and 1b). Biopsy with transurethral resection of the tumor showed a high-grade pleomorphic sarcoma infiltrating widely in mucosa and muscularis propria. A CT scan of her chest was unremarkable and a bone scan showed no evidence of metastatic disease in her skeleton. A radical cystectomy plus bilateral pelvic lymphadenectomy, total hysterectomy and anterior vaginectomy were performed with ileal conduit urinary diversion.

**Figure 2:**
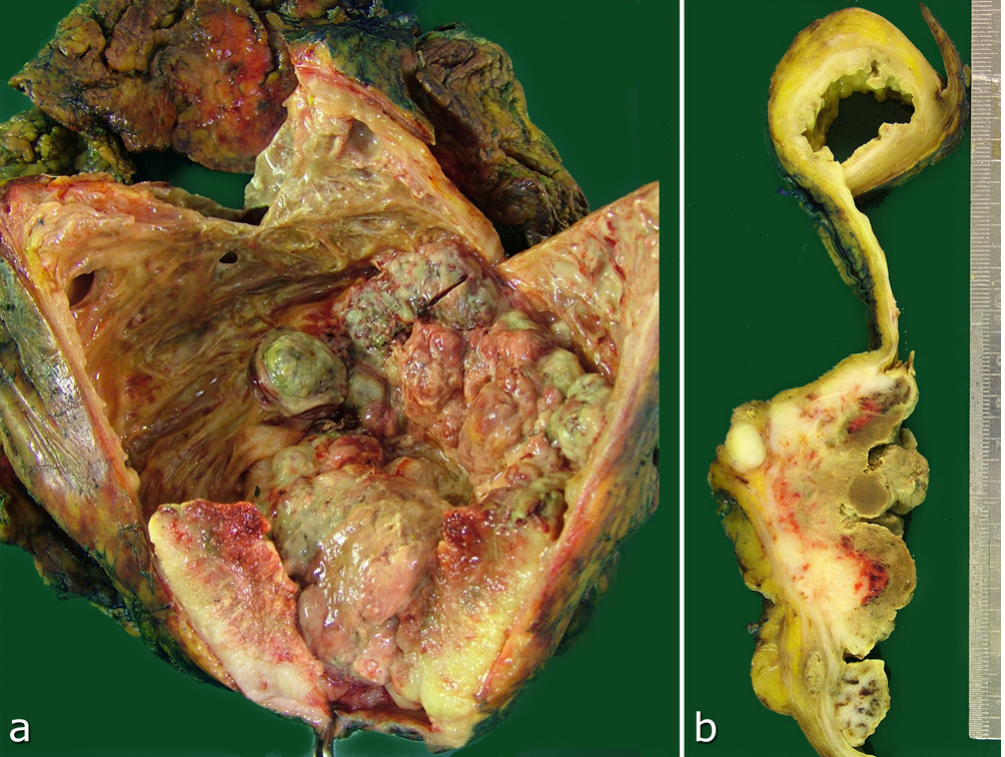
**Macroscopic appearance of the tumor; (b) shows a horizontal slice taken from the body of bladder**.

**Figure 3:**
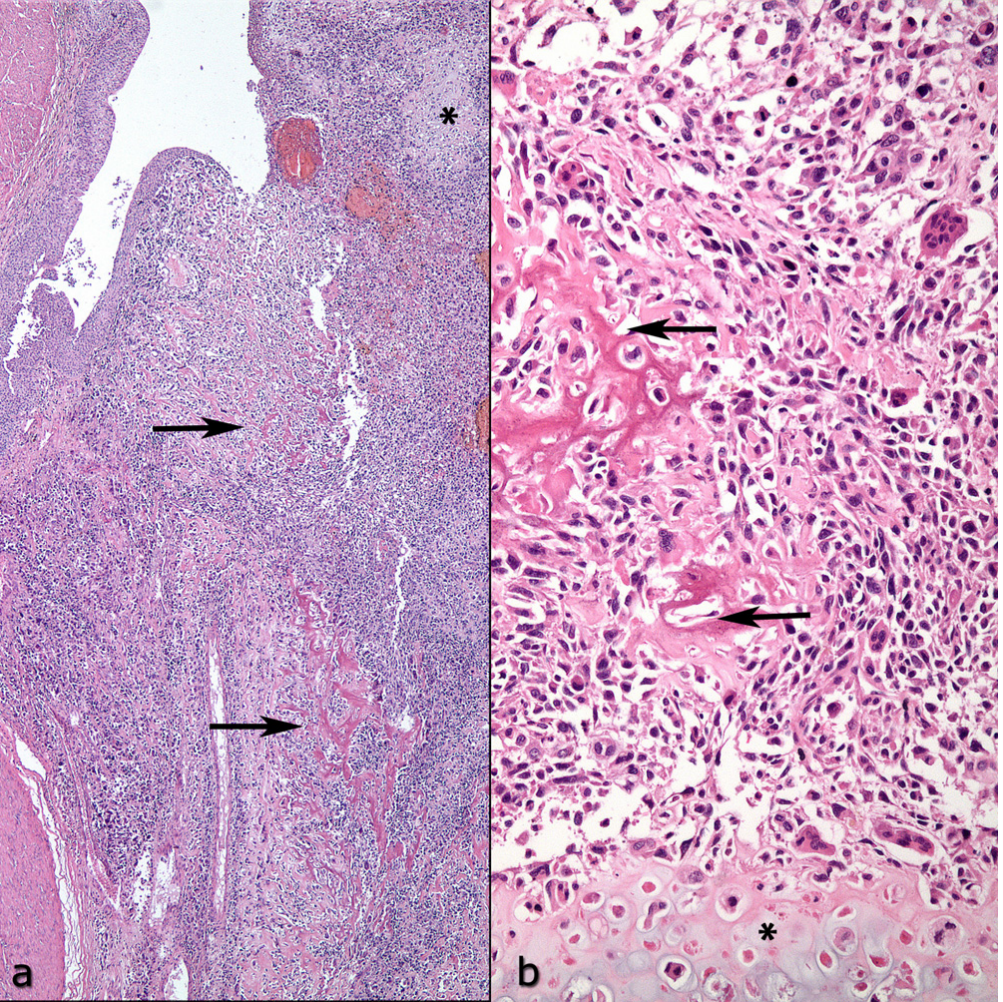
**Low (a) and high power (b) pictures of the sarcoma**. Highly pleomorphic cellular tumor is seen with areas of osteoid (indicated by arrow) and chondroid (indicated by asterix) formations. Non-neoplastic surface epithelium overlying the tumor is apparent in part (a). Osteoclast-type multinucleated giant cells are seen scattered among malignant cells, seen in part (b). (a: H-E × 40; b: H-E × 200).

**Figure 4:**
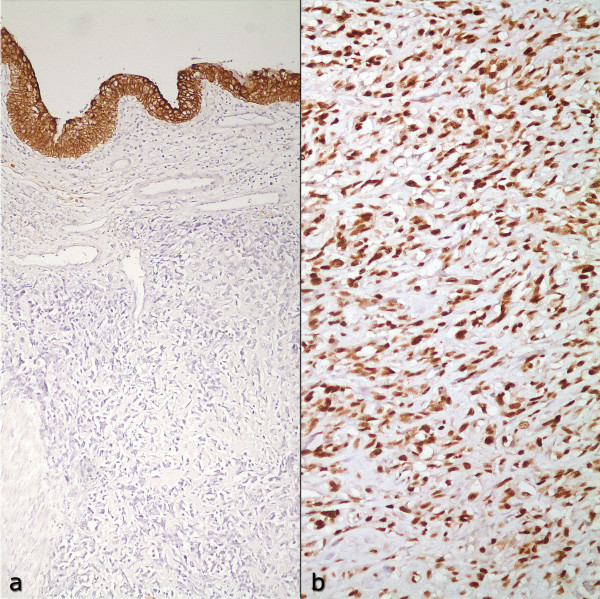
**Immunohistochemistry**. a) Neoplastic cells do not express cytokeratins. Normal urothelium constitutes the positive intrinsic control of the stain (Primary anti-pancytokeratin Ab, ABC × 100). b) Diffuse and strong p53 positivity by the neoplasm (Primary anti-p53 Ab, ABC × 200).

On macroscopic examination of the radical cystectomy specimen, a large ulcerating exophytic polypoid nodular tumor, mainly located on the left lateral and posterior walls of the urinary bladder, was observed. It extended from the dome down to the urethra, involving the trigone and bladder neck plus both ureteral orifices, as well as the right lateral and anterior walls distally (Figure 2). It was a shiny gray to white infiltrative firm mass with areas of softening and necrosis. The tumor had invaded the whole thickness of the bladder wall and penetrated into the perivesical fat tissue, as well as the anterior wall of the vagina. The rest of the bladder mucosa was hemorrhagic and ulcerated. Microscopically, a highly cellular neoplasm composed of pleomorphic spindle or plump cells with a variable amount of eosinophilic cytoplasm was seen. Neoplastic cells formed large areas of malignant cartilage and lacelike osteoid in addition to short interlacing fascicles (Figure 3). There were also occasional osteoclastic-type multinucleated giant cells and the mitotic rate was in excess of 20 per 10 high-power fields. Lymphovascular invasion and large areas of necrosis were also common and the tumor extended to the resection margin at the distal posterior border of the specimen. Immunohistochemical stains performed on the paraffin-embedded material showed no reaction for pan-cytokeratin, cytokeratin 7, cytokeratin 20, p63 or epithelial membrane antigen (EMA) (Figure 4). There was strong p53 nuclear staining in more than 90% of the neoplastic cells and extensive sampling of the rest of the bladder revealed neither papillary urothelial neoplasm nor flat carcinoma in-situ or epithelial dysplasia. The uterus was unremarkable and the bilaterally dissected pelvic lymph nodes showed reactive lymphoid hyperplasia without metastasis. Based on the morphology and negative immunohistochemical staining with epithelial markers, in addition to the clinical absence of another tumor focus elsewhere in the body, a diagnosis of primary osteosarcoma of the urinary bladder was made. There was no evidence of recurrence or metastasis 6 months after surgery.

## Discussion

Primary sarcomas of the urinary bladder are uncommon [1] and most originate from muscle as rhabdomyosarcoma which is dominant in children, whereas leiomyosarcoma is dominant in adults. There are only 30 published cases of primary osteosarcoma of the urinary bladder in the literature [3-5] and they show a male to female ratio of 4:1. The age of the patients ranges from 41 to 86 years (mean 64 years) and the most common presentation is hematuria. The tumors are large, polypoid and deeply infiltrative and the most common single location is the trigone where histology shows a high-grade sarcoma with osteoid production. The differential diagnosis includes many possibilities, including: 1) sarcomatoid variant of urothelial carcinoma; 2) urothelial carcinoma with osseous metaplasia and; 3) carcinoma with pseudosarcomatous stromal reaction. Primary sarcomas of the urinary bladder including leiomyosarcoma, chondrosarcoma, rhabdomyosarcoma, angiosarcoma and malignant fibrous histiocytoma as well as osteosarcoma are much more rare than sarcomatoid urothelial carcinoma. The diagnosis of a sarcoma should only be made after excluding all these possibilities. A prior history of urothelial carcinoma may provide sufficient evidence for a mesenchymal-looking malignancy as being in fact a sarcomatous carcinoma even though there may not be an epithelial component at the time. The immunohistochemical profile of a sarcomatoid carcinoma notably includes positivity for epithelial markers, cytokeratins or epithelial membrane antigen at least focally. However, a positive reaction does not necessarily exclude mesenchymal origin as it is well known that some sarcomas such as malignant fibrous histiocytoma and leiomyosarcoma can co-express epithelial antigens. Furthermore, occasional sarcomatoid carcinoma can be completely negative for any epithelial marker applied. Features that are helpful in making a decision towards carcinoma include identification of nested or clustered epitheloid tumor cells, of either conventional or other types of carcinoma, lying adjacent to sarcomatoid cells. The presence of in-situ carcinoma is also another supporting feature for epithelial origins. In our case, the histology of the bladder tumor was identical to osteosarcoma of the bone with characteristic formation of lace-like osteoid in between malignant cells, as well as an abundant chondroid matrix. No past or accompanying urinary epithelial malignancy was identified; immunohistochemistry did not demonstrate epithelial differentiation and all these findings supported a diagnosis of primary vesical osteosarcoma.

Our current presentation is the first case report of osteosarcoma in a patient with SLE. It has been stated that malignant neoplasms occur more commonly in patients with SLE than in the general population [2]. Cohort studies have yielded varying estimates of the relative cancer risk in SLE, most with fairly wide confidence intervals (CIs). The standardized incidence ratios in these studies ranged from 1.1 (95% CI 0.7–1.6) to 2.6 (95% CI 1.5–4.4) and the risk of non-Hodgkin lymphoma in SLE has been found to be increased 3–4-fold compared with the risk in individuals without SLE [6]. Several types of sarcomas have been observed in SLE, including leiomyosarcoma, Kaposi sarcoma, angiosarcoma and liposarcoma [6-9]. Numerous pathogenic mechanisms have been proposed although hypotheses regarding the specific reasons still remain largely speculative since this issue has not yet been well studied. Patients with SLE have defects in both their cellular and humoral immune systems and prolonged stimulation of B lymphocytes, together with defective immune surveillance, could result in the formation of autonomous B-cell clones and result in lymphoma development. Another possible pathogenic link between SLE and cancer include immunosuppressive treatments.

Our patient had a long history of SLE with her disease being constantly active for 13 years and she was continuously on high dose steroids and cyclophosphamide to achieve immunosuppression. Several groups have described primary leiomyosarcoma in the urinary bladder where patients had been exposed to cyclophosphamide for either neoplastic or non-neoplastic conditions [10,11]. Three of those cases were SLE patients, one of which was Epstein-Barr virus associated.

Cyclophosphamide is a commonly used chemotherapeutic and immunosuppressive medication. It is a direct alkylating agent, activated after intake in the liver by cytochrome P-450, and the associated metabolites are excreted in the urine. The bladder cancer risk is increased 6.8-fold in cyclophosphamide-exposed patients, ranging from 6.4 in the absence of cystitis to 11.3 when hemorrhagic cystitis is present [11,12]. The carcinogenic action is thought to be secondary to urinary excretion of acrolein, one of the metabolites of cyclophosphamide [10-12]. It has been noted that the relative proportion of mesenchymal neoplasms over urothelial malignancies in the urinary bladder is increased with exposure to this drug. Leiomyosarcomas represent 9.2% of bladder tumors in patients exposed to cyclophosphamide compared with 0.1% of sporadic tumors [10].

An additional contributing factor for the increased risk of bladder neoplasia in our case could be the frequent urinary tract infections. The patient suffered from recurrent urinary infections due to Gram negative bacteria. Production of carcinogenic nitrites from urinary nitrates by Gram negative bacteria is highly suspected in the etiology of tumor formation.

The prognosis for vesical osteosarcoma is usually dismal with 22 out of 25 patients dying within 6 months, most as a result of local spread with urinary tract obstruction and secondary infections. Distant metastases are uncommon.

## Conclusion

We recommend that periodic evaluation of SLE patients who have been on heavy immunosuppressive therapy, especially with cyclophosphamide, should include exclusion of malignancies. Particular attention must be paid to the urinary bladder, also taking into account the possibility of uncommon histological tumor types.

## Abbreviations

SLE: systemic lupus erythematosus; EMA: epithelial membrane antigen; CT: computerized tomography;

## Consent

Written informed consent was obtained from the patient for publication of this case report and accompanying images. A copy of the written consent is available for review by the Editor-in-Chief of this journal.

## Competing interests

The authors declare that they have no competing interests.

## Authors' contributions

CH performed the histological examination. HK and HO collected and analyzed the patient data. SY reviewed the literature. DEB was the major contributor in writing the manuscript.
